# Wound of the Abdomen

**Published:** 1829-01

**Authors:** 


### WOUND OF THE ABDOMEN.

### *Punctured Wound of the Abdomen, with Protrusion of a portion of the Omentum.*

William Watson, æt. twenty-one, was admitted at St. Bartholomew's Hospital, under the care of Mr. Earle, on the 8th of November, having a protrusion of omentum, about three inches in length, from a punctured wound on the right side of the abdomen, which he says was inflicted by a carving knife. When admitted, he was sick and faint, and there was a great deal of pain about the parts. The wound was about three-fourths of an inch long, situated in a transverse line from the umbilicus, and about one inch to the outer side of the rectus muscle. The omentum was firmly grasped by the edges of the wound, and was very vascular, being of a vermilion colour: it was bleeding very slightly from its surface. After some unsuccessful attempts had been made to return the omentum, it was deemed advisable to enlarge the wound; but great resistance was still made by the abdominal muscles, and the parts could not be returned without some difficulty. When the omentum was replaced within the abdomen, the wound was closed by one suture through the integuments, care being taken to avoid the peritoneum.

The pulse was rather small, and about eighty. He complained of pain in the wound. Five hours after this, the pulse having risen and become more frequent, and the wound being more painful, the bandage was relaxed, and he was bled to ℥xx. when he fainted. An enema was given, and he had one good evacuation. Four hours after this, the pulse being 108 and full, he was again bled to ℥vi.

9th.—Had a tolerable night; not so much pain in the wound. Pulse ninety, and not very full. Bowels open twice. In the evening the pulse was fuller, but not more frequent, and perfectly compressible; tongue clean.

From this date he gradually improved.

